# Cloning, Expression and Characteristics of a Novel Alkalistable and Thermostable Xylanase Encoding Gene (*Mxyl*) Retrieved from Compost-Soil Metagenome

**DOI:** 10.1371/journal.pone.0052459

**Published:** 2013-01-31

**Authors:** Digvijay Verma, Yutaka Kawarabayasi, Kentaro Miyazaki, Tulasi Satyanarayana

**Affiliations:** 1 Department of Microbiology, University of Delhi South Campus, New Delhi, India; 2 Laboratory for Functional Genomics of Extremophiles, Faculty of Agriculture, Kyushu University, Fukuoka, Japan; 3 Institute for Hearth Research, National Institute of Advanced Industrial Science and Technology (AIST), Hyogo, Japan; 4 Bioproduction Research Institute, National Institute of Advanced Industrial Science and Technology (AIST), Hokkaido, Japan; 5 Department of Medical Genome Sciences, Graduate School of Frontier Sciences, The University of Tokyo, Hokkaido, Japan; Radboud University Medical Centre, NCMLS, The Netherlands

## Abstract

**Background:**

The alkalistable and thermostable xylanases are in high demand for pulp bleaching in paper industry and generating xylooligosaccharides by hydrolyzing xylan component of agro-residues. The compost-soil samples, one of the hot environments, are expected to be a rich source of microbes with thermostable enzymes.

**Methodology/Principal Findings:**

Metagenomic DNA from hot environmental samples could be a rich source of novel biocatalysts. While screening metagenomic library constructed from DNA extracted from the compost-soil in the p18GFP vector, a clone (TSDV-MX1) was detected that exhibited clear zone of xylan hydrolysis on RBB xylan plate. The sequencing of 6.321 kb DNA insert and its BLAST analysis detected the presence of xylanase gene that comprised 1077 bp. The deduced protein sequence (358 amino acids) displayed homology with glycosyl hydrolase (GH) family 11 xylanases. The gene was subcloned into pET28a vector and expressed in *E. coli* BL21 (DE3). The recombinant xylanase (rMxyl) exhibited activity over a broad range of pH and temperature with optima at pH 9.0 and 80°C. The recombinant xylanase is highly thermostable having T1/2 of 2 h at 80°C and 15 min at 90°C.

**Conclusion/Significance:**

This is the first report on the retrieval of xylanase gene through metagenomic approach that encodes an enzyme with alkalistability and thermostability. The recombinant xylanase has a potential application in paper and pulp industry in pulp bleaching and generating xylooligosaccharides from the abundantly available agro-residues.

## Introduction

Hemicellulose represents the second most abundant renewable polymer of plant cell walls after cellulose, and xylan is the main constituent in lignocellulosic agro-residues. The backbone of xylan is mainly composed of β-1,4-linked xylosyl residues along with various groups (arabinosyl, acetyl and glucuronosyl) in their side chains. These heterogeneous polysaccharides play a critical role in maintaining the cell wall integrity by making covalent and non-covalent bonds with cellulosic fibres and lignins [1], [2]. The heterogeneity in xylans necessitates multiplicity in the xylanolytic enzyme system that comprises endoxylanase (1,4-β-D-xylan xylanohydrolase; EC 3.2.1.8), β xylosidase (1,4 β-D-xylan xylohydrolase; EC 3.2.1.37) α-glucuronidase, α-L-arabinofuranosidase, and acetyl xylan esterase. The CAZY database (http://www.cazy.org/fam/acc_GH.html) categorized xylanases into six glycosyl hydrolase families GH5, GH8, GH10, GH11, GH30 and GH43 [3]. Among these, family 10 and 11 xylanases are widely distributed where GH10 xylanases share versatile substrate specificity with higher molecular weight, while GH11 xylanases are more stringent in their substrate specificity with low molecular weight and are considered as true xylanases [4], [5]. Hydrolysis of xylans by xylanases is of interest to many industries like ramie fibre degumming, food processing, textile, biofuel, feed and paper/pulp industries [6], [7], [8]. The xylanases that withstand extreme conditions prevailing in the industrial processes are in high demand in paper pulp processing and feed industries.

Although several xylanases have been reported from a number of microorganisms, most of them do not have adequate thermostability and alkalistability for their application in paper and pulp industries. Majority of xylanases have been obtained from the culturable 0.1 to 1% of the total microbial diversity existing in natural environments. The culture-independent metagenomic approaches permit retrieval of genes encoding useful enzymes from environmental samples without involving laborious and elaborate methods for the cultivation of microbes. The immense demand for alkalistable and thermostable xylanases encouraged us to adapt this innovative strategy for retrieving genes that encode thermo-alkali-stable xylanases. In this investigation, a metagenomic library was constructed and screened for clones with xylanase activity. Xylanase encoding gene (*Mxyl*) was subcloned and expressed, and the recombinant xylanase was purified and characterized. To the best of our knowledge, this is the first report on retrieving thermo-alkali-stable xylanase by a metagenomic approach.

## Results

### Construction of metagenomic library, DNA sequencing and bioinformatics analysis

**Figure 1:**
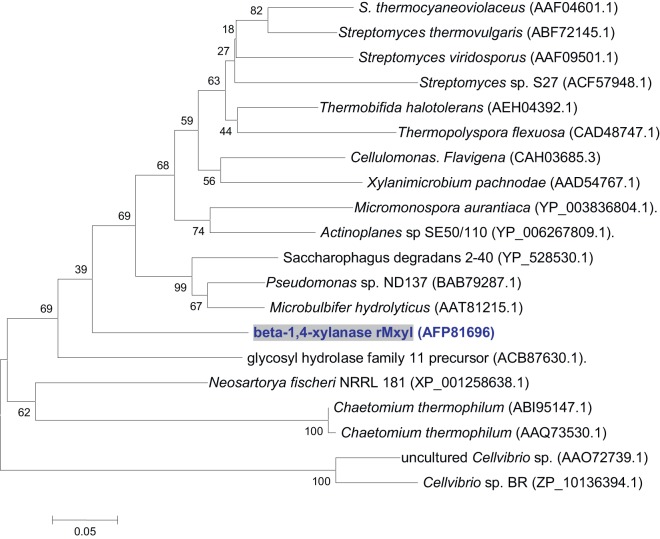
Phylogenetic tree of recombinant xylanase. rMxyl showed highest homology with xylanase of *Cellulomonas fimi* ATCC 484 followed by uncultured microbial GH 11 xylanase. Neighbour Joining (NJ) Tree is constructed by using MEGA 4.0 software. Bootstrap values (n = 1000 replicates) are represented as percentage. The scale bar depicts the allowed changes per amino acid position.

When 5.0 µg of 20–30 kb of high molecular weight metagenomic DNA was digested with Sau3AI and the fragments were ligated into p18GFP vector with an efficiency of 3.6×10^4^ clones per µg of DNA in constructing the library, the insert sizes were in the range of 3.0–8.0 kb with an average size of 5.5 kb. On screening, a clone having xylanase gene was spotted on RBB xylan containing LB-amp plate. The full sequence of the insert showed the size of 6.231 kbp that revealed its prokaryotic origin on blast analysis. The complete insert contained nine transcriptional units with a complete ORF of 1077 bp long xylanase gene. The sequence showed putative sequences of -35 (CACGCCA), -10 (TAAAAA) and ribosomal binding sites (AGGGG) at the upstream of xylanase gene followed by complete ORF having ATG and TAA as start and stop codons, respectively (Figure S1). The xylanase displayed five conserved regions (I-V) of GH11 xylanase having two catalytically important residues (Glu_109_ and Glu_217_) present in signature sequence II and V (Figure S2). Amino acid homology showed maximum identity (79%) with the xylanase gene of an uncultured bacterium and *Actinoplanes* sp. SE50/110 followed by a metagenomic GH 11 xylanase (71%). It shared 63–75% homology with xylanases produced by various species of *Streptomyces*. The xylanase retrieved in this investigation exhibits 75, 67 and 64% similarity with the endo-1,4 β-xylanases of *Cellulomonas fimi*, *Micromonospora aurantiaca* 27029 and *Amycolatopsis mediterranei* U32, respectively. It, however, has lower homology with the xylanases of *Microbulbifer hydrolyticus* (63%), *Pseudomonas* sp. ND137 (62%), uncultured *Cellvibrio* sp. (58%), *Cellvibrio mixtus* (57%) and *Aspergillus fumigatus* AF293 (52%) (Figure 1).

### Construction and expression of the xylanase gene in *E. coli* and localization of the encoding recombinant xylanase (rMxyl)

Xylanase gene was successfully cloned into pET28a(+) vector. It was confirmed by the release of insert from the vector on double digestion with NheI and XhoI. The sequencing results further confirmed the insert. The recombinant plasmid was expressed in *E. coli* BL21 (DE3) on induction with 1.0 mM IPTG at A_600_ of 0.6–0.7 and 30°C. At higher level of expression, it led to the formation of inclusion bodies, which could be solubilized using 6.0 M urea. The highest titre of the recombinant enzyme was achieved in 4–6 h. The construct (*pET28a-Mxyl*) expressed a high proportion of xylanase in cytoplasmic fraction (83%), followed by periplasmic (9%) and extracellular (8%) fractions after 4–5 h of induction. When xylanase gene was cloned and expressed in pET22b(+) vector, a high proportion of intracellular enzyme (>60%) was produced in the initial 3 h of induction, and thereafter, it declined. The periplamic xylanase was optimum at 12 h, while the extracellular fraction gradually increased and it reached a peak (29%) in 24 h.

### Site directed mutagenesis

Muteins having Glu_117_Asp and Glu_209_Asp completely lost the activity. These two glutamates are highly conserved residues in the signature sequences LV**E**YYIVDN and MAT**E**GY, and these are responsible for catalytic activity of GH 11 xylanase.

### Purification, biochemical characterization and zymogram analysis of rMxyl

**Figure 2:**
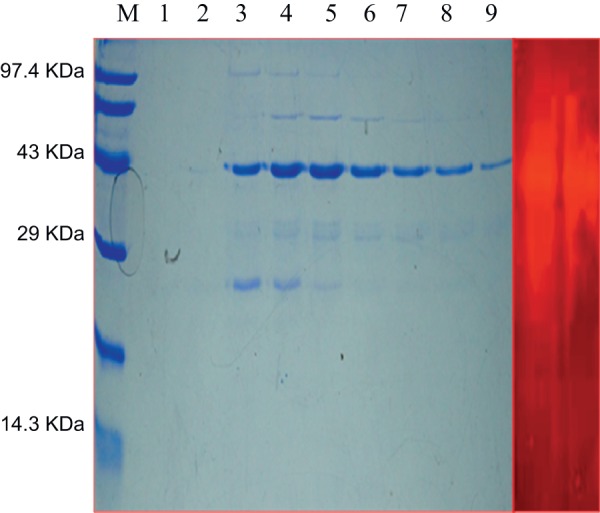
Analysis of rMxyl using SDS–PAGE (15% polyacrylamide gel). **A.** Lane 1 protein marker, Lane 2 and 3 are washes with 20 and 30 mM mM imidazole. Recombinanat xylanase was eluted using different concentrations of imidazole (100, 200, 250, 300, 400, 450, 500 mM). Purified xylanase showed molecular mass of ∼42 kDa on staining with Coomassie Brilliant Blue R-250. **B.** Zymogram analysis of purified xylanase using Congo red staining method.

**Figure 3:**
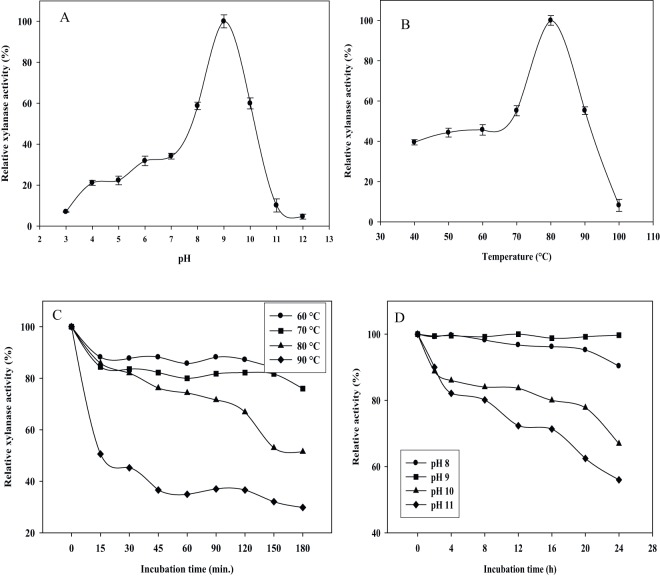
Effect of pH and temperature on the activity and stability of rMxyl. **A** and **B:** The recombinant xylanase incubated in various buffers (pH 3–12) and temperatures (40–100°C) and assayed for xylanase activity **C:** Recombinant xylanase was incubated in glycine-NaOH buffer without substrate and kept at various temperatures. Aliquots were collected at various time interval and store at 0°C for calculating residual activity. **D:** Similarly enzyme was incubated in various buffers (pH 8–11) and aliquots of different time intervals were used xylanase assays.

The recombinant xylanase was purified by Ni^2+^-NTA resin affinity chromatography and the purified recombinant protein could be eluted using imidazole (100–400 mM). The protein appeared as a single band of 40 kDa against the protein marker on 15% SDS-PAGE, and the recombinant xylanase revealed as a clear band of xylan hydrolysis by zymogram analysis (Figure 2). The effects of various physical and chemical parameters on the recombinant xylanase have been assessed. The xylanase exhibited broad range of pH (6.0–12.0) with optimum at 9.0, and it retained ∼55% residual activity at pH 10.0 (Figure 3A). The rMxyl is active in the temperature range between 40 and 100°C (Figure 3B) with optimum at 80°C, and retains more than 90–95% activity after exposure to 60 and 70°C for 3 h. The enzyme has a T_1/2_ of 2.0 h at 80°C and 15 min at 90°C (Figure 3C). The recombinant enzyme did not lose activity after 3 h exposure to pH 8.0 and 9.0, and thereafter, it declined (50% residual activity after 4 h). Approximately 20–45% loss in activity was recorded on either side of the pH optimum after 1 h incubation (Figure 3D). Mg^2+^, Sn^2+^ and Fe^2+^ stimulated rMxyl activity, while Hg^2+^ and Mn^2+^ significantly inhibited enzyme activity even at 1 mM. Other metal ions exerted inhibitory action on xylanase. More than 30% activity was lost in the presence of Mn^2+^ (Table 1). NBS and PMSF inhibited the activity to a significant extent even at 1 mM concentration. β-ME and DTT strongly inhibited enzyme activity. A stimulatory effect EDTA was recorded on xylanase activity.

**Table 1:** Effect of modulators on rMxyl activity.

Metal ions	1 mM	5 mM	10 mM
Mg^2+^	106.45±1.05	99.65±0.98	87.38±0.45
Fe^2+^	108.65±0.75	116.01±0.27	93.67±1.32
Sn^2+^	110.43±0.67	76.12±0.44	45.17±0.63
Ni^2+^	91.21±0.22	79.01±1.34	32.84±0.43
Zn^2+^	91.67±0.32	76.64±0.78	32.89±0.89
Pb^2+^	81.33±067	20.78±0.32	09.65±0.67
K^+^	81.21±1.08	20.62±0.12	12.67 ±0.45
Ag^2+^	73.48±0.53	54.55±0.69	27.83±0.98
Ca^2+^	72.43±0.43	35.45±0.21	12.09±0.19
Mn^2+^	71.76±0.63	27.34±1.32	09.67±0.27
Ba^2+^	66.45±0.67	23.91±0.34	18.65±0.33
Cd^2+^	54.67±0.43	29.33±0.49	12.87±0.65
Co^2+^	59.15±1.23	29.63±0.65	12.54±1.12
Na^+^	61.43±0.78	39.75±1.06	27.35±0.78
Cu^2+^	29.12±0.18	15.76±0.76	10.09±0.87
Hg^2+^	0	0	0
**Inhibitors**	**1 mM**	**5 mM**	**10 mM**
NBS	46.66±0.12	35.67±0.09	20.12±0.11
IAA	103.45±0.54	89.75±0.32	69.85±1.56
β-ME	0	0	0
DTT	0	0	0
EDTA	105.65±1.23	107.19±1.01	89.98±0.56
**Detergents**	**0.1% (v/v)**	**0.5% (v/v)**	
Tween 20	103.45±1.32	105.67±0.98	
Triton X100	108.32±0.96	104.05±0.92	
SDS	97.34±1.32	65.89±0.19	
Control	100±0.12	100±0.23	100±0.67

Most of the metal ions were insignificant at 1 mM concentration; however xylanase activity was significantly inhibited at higher concentration by Pb^2+^, Ag^2+^, Ca^2+^, Mn^2+^, Ba^2+^, Cd^2+^ and Co^2+^. In the presence of Hg^2+^, enzyme lost completely its activity. Similarly, trace amounts of β-mercaptoethanol (β-ME) and dithiothreitol (DTT) completely inhibited the xylanase activity. Inhibition in the presence of N-bromosuccinimide (NBS) signifies the role of tryptophan in catalysis, while EDTA confirms it as a non-metallozyme.

### Saccharification of agroresidues/hydrolysis of xylan

**Figure 4:**
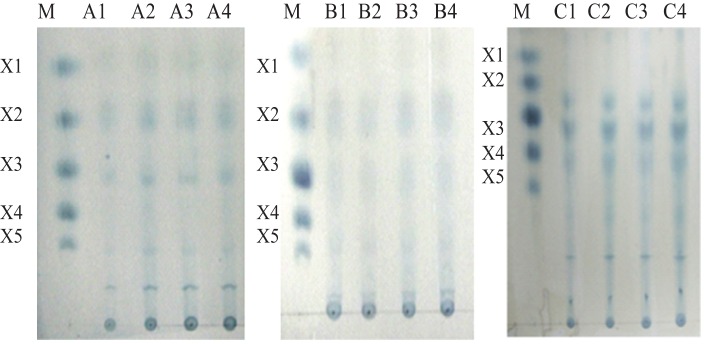
Profile of xylooligosaccharides liberated by the action of rMxyl. Lane (A1–A4)*: spots of X1, X2 and X3 were detected from wheat bran. Lane (B1–B4)*: hydrolysis from corncob that showed prominence of X2 and X3. While, X3, X4 and X5 were detected from hydrolysate of sugarcane bagasse (C1–C4)*. Lane M: Standards of various XOs (Sigma, USA). X1: xylose, X2: xylobiose; X3: xylotriose; X4: xyloptetraose; X5: xylopentaose *: 1/2/3/4 time intervals of 5, 15 and 30 min and 1 h, respectively.

The rMxyl hydrolyzed xylan from various sources. The enzyme activity was very high in birchwood xylan (relative activity 100%) in comparison with that on xylan from beech wood (97%) and arabinoxylan (80%). There was no activity on carboxymethylcellulose (CMC) and other non-xylan polysaccharides (starch, pullulan and chitin). The K_m_ and V_max_ values of the enzyme on birchwood xylan are 8.0±1.21 mg/ml and 300±09.12 μmol/min/mg, respectively. The saccharification of wheat bran was high (15.2%) as compared to that of corncobs (9.89%) and sugarcane bagasse (4.71%). Various xylooligosaccharides were detected in the hydrolysates (Figure 4).

## Discussion

Although several xylanases have been reported from diverse microbiota using traditional culture dependent approaches [9], [10], [11], [12], majority of them do not endure the extreme temperature and alkaline conditions prevailing in industrial processes. An alternate strategy was, therefore, adapted to retrieve a thermo-alkali-stable xylanase gene (*Mxyl*) by culture-independent metagenomic approach. The metagenomic library constructed with DNA extracted from the compost-soil samples yielded a clone that produced xylanase. Although, the compost soils are in the acidic pH range, an alkalistable and thermostable endoglucanase had been reported from rice straw compost [13]. The culture independent approach has started yielding the useful biocatalysts from the hidden Pandora's Box [14], [15], [16]. A considerable success has also been achieved in obtaining xylanases with diverse attributes by using metagenomic approaches [17], [18], [19], [20], [21]. The protein encoded by xylanase gene comprises 358 amino acids, of which 16 are acidic and 21 basic. The predicted molecular weight, pI and instability index of recombinant xylanase are ∼40 kDa, 8.8 and 33.44 respectively. The xylanase contained a 43 amino acid long leader sequence at the N terminal region followed by a catalytic domain (44^th^–212^th^) of GH11 family interrupted by a short stretch of arginine and threonine rich non-catalytic region (WSVRQ_2_R_2_TG_2_TIT_2_). In addition, serine rich Q linker region (S_2_GS_2_DITVG_2_TS_2_G_2_TS_2_G_2_S_3_G_2_S_10_G_4_) has also been detected from amino acid 213 to 248 just after catalytic domain. Such repeated amino acids make linker regions that usually discriminate catalytic domain from carbohydrate binding domain [22], [23], [24]. Moreover, linkers have also been reported as integral parts of various xylanases that connect thermo-stabilizing domains, surface layer homology domains and dockerin domains which play a role in stabilizing the protein [22], . Amino acid homology and hydrophobic cluster analysis categorized this high molecular weight xylanase into GH 11 family. This is not unusual because xylanases of *Clostridium stercorium* also had been classified as a GH11 xylanase despite its high molecular mass (56 kDa) [29]. Metagenomic origin, distinct characteristics, lower homology and higher molecular weight (>30 kDa) make this a novel xylanase. The recombinant xylanases from *Glacieola mesophila* KMM241 and *Geobacillus thermoleovorans* displayed a similar pattern of recombinant protein localization [30], [31]. The profile of the distribution of the recombinant xylanase is similar to other xylanases cloned in pET28a(+) and pET22b(+) vectors as in *G. thermoleovorans* and *B. halodurans* [30], [32]. The integrated N- terminal pelb signal sequence in pET22b(+) directed the enzyme to periplasm that further led to secretion into the extracellular environment.

The site directed mutagenesis of two residues of glutamate to aspartate resulted in a complete loss of xylanase activity due to disruption in double displacement mechanism [33]. In order to take the advantage of thermostability of the recombinant xylanase, it was subjected to high temperature prior to purification by Ni^2+^-NTA agarose resins. This step reduced the extra load of non-His tagged, less thermostable and contaminant host proteins [30], [32].

The rMxyl exhibits optimum activity at higher temperature and pH which is similar to xylanases produced by *Dictyoglomus thermolacticum*, *Thermotoga maritima*, *Bacillus acidicaldarius* and *Geobacillus thermoleovorans* having optimal activity at or above 80°C [30], [34], [35], [36]. The activity and stability of rMxyl at higher pH are the crucial properties of xylanases for their applicability in paper processing industry. Xylanases of *G. thermoleovorans*, *Bacillus firmus*, *B. stereothermophilus* T-6 exhibited optimum activity at pH at 8.0 or above [30], [37], [39]. The shelf life of rMxyl is more than 3 months at 4°C, which retains greater than 90% activity. The recombinant xylanase is optimally active at 80°C and pH 9.0 that distinguishes it from already reported xylanases. The xylanase of *Thermotoga maritima* has T_opt_ of 90°C, but it gets inactivated fast at pH 6.0 [35]. The highly thermostable xylanase with optimum temperature in the range between 80 and 105°C are available, but these xylanases exhibit their maxima either at acidic or neutral pH [35,35]. Similarly the alkalistability at higher pH is reported in many xylanases but are active at lower temperatures [37], [38]. The recombinant xylanase of GH10 family from *Bacillus halodurans* showed both properties together having optima at 75°C and pH 9.0, but it losses 50% activity at 65°C after 4 h and gets inactivated very fast at 80°C [32]. The metagenomic xylanase, on the other hand, has good thermostability at higher temperatures (60, 70 and 80°C) with only 20–30% loss after 3 h exposure. The most significant aspect of this investigation is obtaining a highly alkalistable (pH_opt._ 9.0) and thermostable (T_opt._ 80°C) xylanase from environmental samples by a metagenomic approach.

Cations (Mg^2+^, Sn^2+^ and Fe^2+^) stimulated the rMxyl activity like those of *B. subtilis* AMX-4 and an uncultured microbe [39], [40]. Even at 1 mM, Hg^2+^ and Mn^2+^ significantly inhibited the activity. The inhibition of xylanase by Hg^2+^ suggests the presence of tryptophan residues that oxidize indole ring, thereby inhibiting the xylanase activity [39], [41], [42], [43]. Xylanases from *Streptomyces olivaceoviridis* A1, *Streptomyces* sp. S27 and *Bacillus subtilis* strain R5 had been reported to be stimulated by Fe^2+^ and Mg^2+^ [19], [44], [45], [46], [47], and total loss of the enzyme in presence of Hg^2+^ and Mn^2+^ was reported in the xylanases from an uncultured microbe [19], [46], *Penicillium* sp. [48] and *Plectosphaerella cucumerina* [41]. The inhibition of xylanase activity by Cu^2+^ is similar to the majority of the xylanases [13], [39], [45], [49]. The stimulatory effect of EDTA could be due to a different catalytic mechanism as reported in xylanases of *Aspergillus niger* and *Fusarium proliferatum* [42], [49]. In *Glaciecola mesophila* KMM 241, EDTA caused ∼25% enhancement in activity [31]. N-BS inhibition suggests the involvement of tryptophan in xylanase activity [50], [51], [52]. Total loss of xylanase activity by β-ME and DTT suggests the distortion of disulfide linkages present between cystein residues [41], [49]. Detergents exerted a slight stimulatory effect on the recombinant xylanase which is a common feature of the other xylanases. However, rMxyl was inhibited by SDS as in other xylanases [39], [46], [49].

The rMxyl hydrolyzed birchwood and beechwood xylan efficiently. The structural similarity of beechwood and birchwood xylans may be the reason for the high activity [31]. The enzyme exhibited almost similar activities on oat spelt and arabinoxylan. Oat spelt xylan is a type of arabinoxylan very rich in arabinose (xylose/arabinose  = 66∶34) [53], [54]. Interestingly the rMxyl liberated xylooligosaccaharides from xylan in just 5 min and it was sustainable on prolonged incubation. Several xylanases have been reported from various microorganisms that liberate xylooligosaccharides following xylan hydrolysis. Alkaline xylanases show better action on agro-residues by lowering the steric hindrance caused by cellulose and enhancing the solubility of hemicellulosic materials [54]. Xylanases from *G*. *thermoleovorans* [30], *Bacillus halodurans* [55] *Thermomonospora fusca* [56] have been reported to generate a similar profile of XOs. The metagenomic xylanase finds application in food industry for the production of xylooligosaccharides as prebiotics [57].

## Conclusions

Most of the xylanases retrieved by culture-dependent and culture-independent approaches exhibit optimum activity in the pH and temperature range of 6.0–8.0 and 40–60°C, respectively. The xylanase obtained in this investigation through metagenomic approach (rMxyl) not only displays alkalistability and thermostability, but it also has a high thermostability. This is the first report of xylanase with twin stabilities obtained through culture-independent approach. A very low similarity in amino acid sequence of the enzyme with other known xylanases makes it a novel xylanase. The possibility of obtaining thermo-alkali-stable xylanase from composts may lead to an intense search for similar enzymes in this niche.

## Materials and Methods

### Ethics statement

No specific permits are required for collecting environmental samples used in this investigation. The samples did not have any animal or plant species because of the elevated temperature. The location is not privately owned or protected in any way and does not involve endangered and protected species.

### Sample collection

The samples of compost-soil were collected in sterile polyethylene bags from the vicinity of a hot water spring near Fukuoka Japan and stored at 4°C. The pH of the samples is in the acidic range (3.0–4.5).

### Strains and plasmids for DNA manipulations

Cloning vector p18 GFP was the gift from Dr. Taku Uchiyama, Japan. The pGEM-T Easy vector (Promega, USA), pET28a(+) and pET22b(+) of Novagen (Germany) were used for sequencing and expression of the xylanase gene, respectively. *Escherichia coli* DH10B, *E. coli* XL1 blue and *E. coli* BL21 (DE3) were used for the propagation of the plasmid. Restriction enzymes and T4 DNA ligase were purchased from New England Biolabs (UK).

### Construction of metagenomic library

Soil DNA was extracted according to Verma and Satyanarayana (2011) [58]. Metagenomic DNA was processed for constructing the metagenomic library. Five μg of metagenomic DNA was partially digested with 0.5 U of restriction enzyme Sau3AI. The fragments of 3–12 Kb were eluted from agarose gel (1.2%, w/v) by gel extraction kit according to manufacturer's protocol (Macherey-Nagel, Germany). Hundred nanogram (ng) of insert DNA and 300 ng of BamH I digested and dephosphorylated p18 GFP vector were ligated by using T4 DNA ligase overnight at 16°C. The ligation mixture was transformed into competent *E. coli* DH10B cells by heat shock method. The metagenomic library was spread and screened for xylanase activity on 0.3% (w/v) RBB-xylan (4-O-methyl-D-glucurono-D-xylan-remazol brilliant blue R) (Sigma, St. Louis, MO, USA) LB-ampicillin agar plates. The transformants were grown at 37°C overnight and observed for the zone of xylan hydrolysis.

### DNA sequencing and bioinformatics analysis

The pure clone (TSDV-MX1) showing clear zone of xylanase hydrolysis was sequenced using M13 forward and reverse primers followed by different internal primers using Applied Biosystem 373 stretch automated sequencer (Applied Biosystems, Foster City, CA, USA) at Nucleic acid sequencing facility of the University of Delhi South Campus, New Delhi (India) for obtaining full sequence of the insert. The ORFs were identified by using the NCBI's open reading frame (ORF) Finder tool (http://www.ncbi.nlm.nih.gov/gorf/gorf.html). BLASTN and BLASTP of NCBI were used to align the nucleotide and amino acid sequences, respectively. Multiple alignments of the amino acids were carried out using the CLUSTALW programme (http://www.ebi.ac.uk/clustalW). The phylogenetic analysis was done using MEGA 2.1 with neighbour joining strategy.

### Construction of expression plasmids *pET28a-Mxyl* and *pET22b-Mxyl*

The full length xylanase gene was pulled out from the clone (TSDV-MX1) by PCR using specific primers MxylF1 (CCCGCTAGCATGACAGCGAGTTTGAGGAAGA) and MxylR1 (CCCCTCGAGTTACGGCGTGTTTCCGTAGC) having the compatible NheI and XhoI restriction sites of pET28a(+) in the forward and reverse primers, respectively. Mxyl F2 (CCCGGATCCATGACAGCGAGTTTGAGGAAGA) and Mxyl R2 (CCCCTCGAGTTAC-GGCGTGTTTCCGTAGC) were used for cloning into pET22b(+) vector. The gene was amplified under the defined PCR conditions (initial denaturation 3 min at 95°C followed by 29 cycles of 10 sec at 98°C, 30 s at 59°C and 1 min at 68°C with a final extension step at 68 °C for 10 min). The PCR products pET28a(+) and pET22b(+) were digested with the restriction enzymes and ligated into the vectors followed by transformation into competent *E. coli* XL1 blue cells to obtain *pET28-Mxyl and pET22-Mxyl*.

### Expression of xylanase (rMxyl) using *pET28a-Mxyl* and *pET 22b-Mxyl*

The recombinant constructs were confirmed by colony PCR followed by double digestion of the construct with restriction enzymes. These clones were processed for sequencing. The recombinant plasmid having the accurate sequence was then transformed into *E. coli* BL21 (DE3) competent cells for expression of the recombinant protein. The transformants were grown in kanamycin (50 µg/ml) containing LB medium at 37°C with 200 rpm in an incubator shaker to achieve an optical density of O.D.  = 0.5 to 0.7. Afterwards the expression was induced by adding isopropyl-β-D-1-thiogalactopyranoside (IPTG) to a final concentration of 1 mM and the culture was further cultivated at 30°C. The samples were collected at 1 h intervals for determining the enzyme titres.

### Site directed mutagenesis

Multiple sequence alignment of recombinant xylanase with those of the known xylanases revealed Glu_117_ and Glu_209_ as catalytically important residues. Experimentally it has been proved by site directed mutagenesis using Geneart site directed mutagenesis kit (Invitrogen, Carsband, USA). Two point mutations (Glu_117_Asp and Glu_209_Asp) were created in the native xylanase gene and expressed in *E. coli* BL21 (DE3). The induced mutations were confirmed by sequencing.

### Localization of rMxyl

Localization of the recombinant protein was determined by collecting the intracellular, extracellular and periplasmic fractions from the same culture. Extracellular, intracellular and periplasmic fractions were collected according to Verma and Satynarayana, 2012 [30].

### Xylanase assay

Xylanase was assayed according to Archana and Satyanarayana (1997) [59]. The 0.5 ml of appropriately diluted enzyme was incubated at 80°C with 0.5 ml of 1% (w/v) birchwood xylan (Sigma, St. Louis, Mo.) in 100 mM glycine-NaOH buffer (pH 9.0) for 10 min. The reducing sugars were determined using 3, 5-dinitrosalicylic acid (DNSA) reagent [60]. One unit of xylanase is defined as the amount of enzyme required to liberate 1 μmol of reducing sugar as xylose ml^−1^ min^−1^ under the assay conditions.

### Purification of recombinant xylanase and zymogram analysis

The rMxyl was purified by affinity chromatography using Ni^2+^-NTA agarose (Novagen, Germany) according to Verma and Satynarayana, 2012 [30]. For zymogram analysis, the recombinant protein was electrophoresed on native gel and the stripes of the gel were layered on 0.3% (w/v) xylan-agar plates of pH 9.0 at 70°C for 3 h. The gel was then removed from the plate and stained with Congo-red dye followed by destaining with 1 M NaCl for better appearance of the band showing clear zone of xylan hydrolysis.

### Biochemical characterization of rMxyl

The kinetic characteristics of the recombinant xylanase like the effect of pH, temperature, metal ions, inhibitors and detergents on enzyme activity, thermostability and substrate specificity have been studied. The optimum pH was determined by incubating the recombinant enzyme in various buffers ranging from pH 3.0 to 12.0. Different buffers were used (pH 3.0 to 6.0, 0.1 M citrate buffer, for 7.0 and 8.0, 0.1 M sodium phosphate buffer and for 9.0–12.0, glycine-NaOH buffer). Similarly for optimum temperature, the recombinant enzyme was assayed over a range of temperatures from 40 to 100°C at pH 9.0. For thermostability, the residual xylanase activity was measured after incubating the recombinant enzyme at different temperatures (60–100) °C for 15 min to 3 h. The effects of various metal ions, inhibitors and detergents were determined by incorporating these into reaction mixtures followed by xylanase assay. The substrate specificity was quantitated by assaying the recombinant enzyme on various substrates (different sources of xylan as well as substrates other than xylan like starch, cellulose, chitin and pullulan in glycine-NaOH buffer). Kinetic properties of the recombinant enzyme (K_m_ and V_max_) on different xylans from birchwood, beechwood and oat spelt were calculated from Lineweaver-Burk double reciprocal plots.

### Saccharification of agroresidues/hydrolysis of xylan

One % (w/v) standards of xylooligosaccharides (X2–X6) and agro-residues (wheat bran, corn cobs and sugarcane bagasse) were treated with recombinant xylanase (10 U – 20 U/g) to find out the hydrolysis of XOs and lignocellulosic substrates. All the substrates (wheat bran, corn cobs and sugarcane bagasse) were suspended in glycine-NaOH buffer (pH 9.0) and incubated at 80°C. Aliquots at the desired intervals were collected and analyzed on silica based TLC plates (Merck, Germany) to determine the hydrolysis products. The saccharification of agro-residues was determined using DNSA reagent [60].

### Nucleotide sequence accession numbers

The nucleotide sequence of the xylanase gene is deposited at NCBI GenBank (Accession no. AFP81696).

## Supporting Information

Figure S1 **Deduced amino acid sequence of recombinant xylanase (rMyl) and its nucleotide sequence.** The red underlined region is leader sequence, cyan highlighted regions represents GH11 catalytic domain. Grey highlighted regions are compositionally biased regions that were not used in database search and proposed as linker regions. Bluish-green highlighted region depicts substrate binding domain.
(DOC)

Figure S2 **Multiple sequence alignment of xylanase with other xylanases available in database.** GenBank accession number and source of microorganisms were given as follows: 182406872 (glycosyl hydrolase family 11 precursor [uncultured bacterium]),17826947 (*Pseudomonas* sp. ND137), 29367333 (uncultured *Cellvibrio* sp.), 388259220 (*Cellvibrio* sp. BR), 302868167 (*Micromonospora aurantiaca* ATCC 27029), 386849796 (*Actinoplanes* sp. SE50/110), 194368056 (*Streptomyces* sp. S27). Five signature sequences: **I** (AYLTLYGW) **II** (VEYYIVDN), **III** (FWQYWSV), **IV** (HFDAWASLG) and **V**(MATEGY) of GH11 family are coloured. The two catalytically important residues (**Glu 117** and **Glu 209**) are marked with black circle.
(DOC)
