# Supplementary figures and images for: Cloning, Expression and Characteristics of a Novel Alkalistable and Thermostable Xylanase Encoding Gene (Mxyl) Retrieved from Compost-Soil Metagenome
Source: PLoS One. 2013 Jan 31;8(1):e52459. doi: 10.1371/journal.pone.0052459 (PMC3561394; doi:10.1371/journal.pone.0052459)

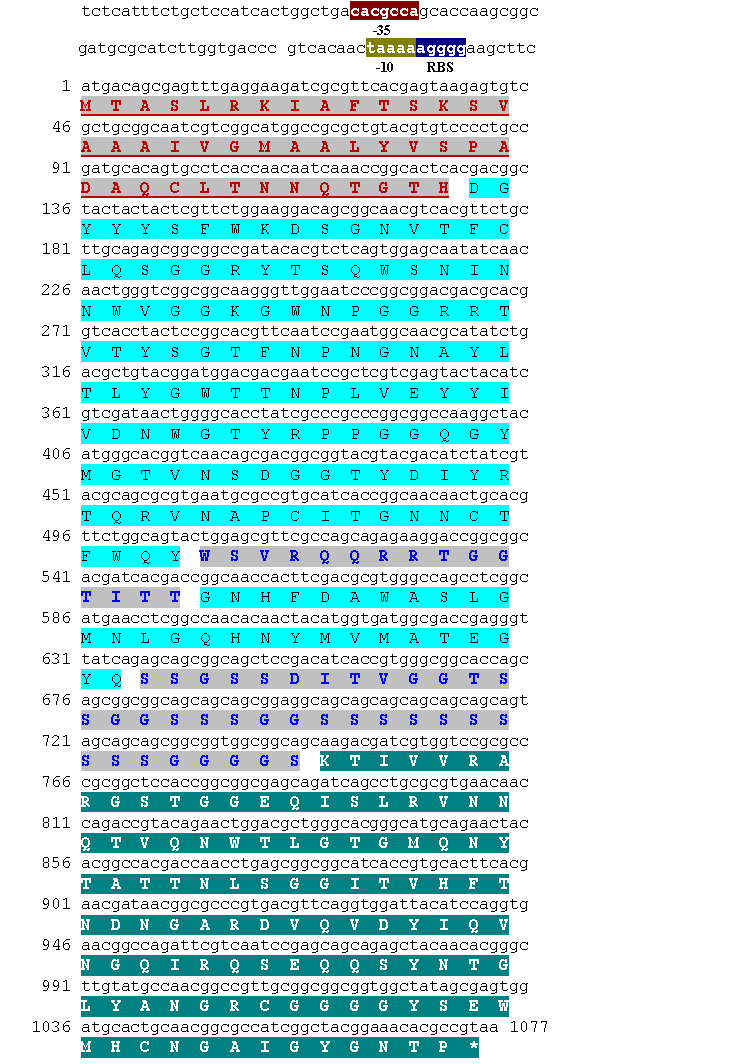


**Figure S1**

Supplement: Figure S1 — Deduced amino acid sequence of recombinant xylanase (rMyl) and its nucleotide sequence. The red underlined region is leader sequence, cyan highlighted regions represents GH11 catalytic domain. Grey highlighted regions are compositionally biased regions that were not used in database search and proposed as linker regions. Bluish-green highlighted region depicts substrate binding domain. (DOC) [file pone.0052459.s001.doc]

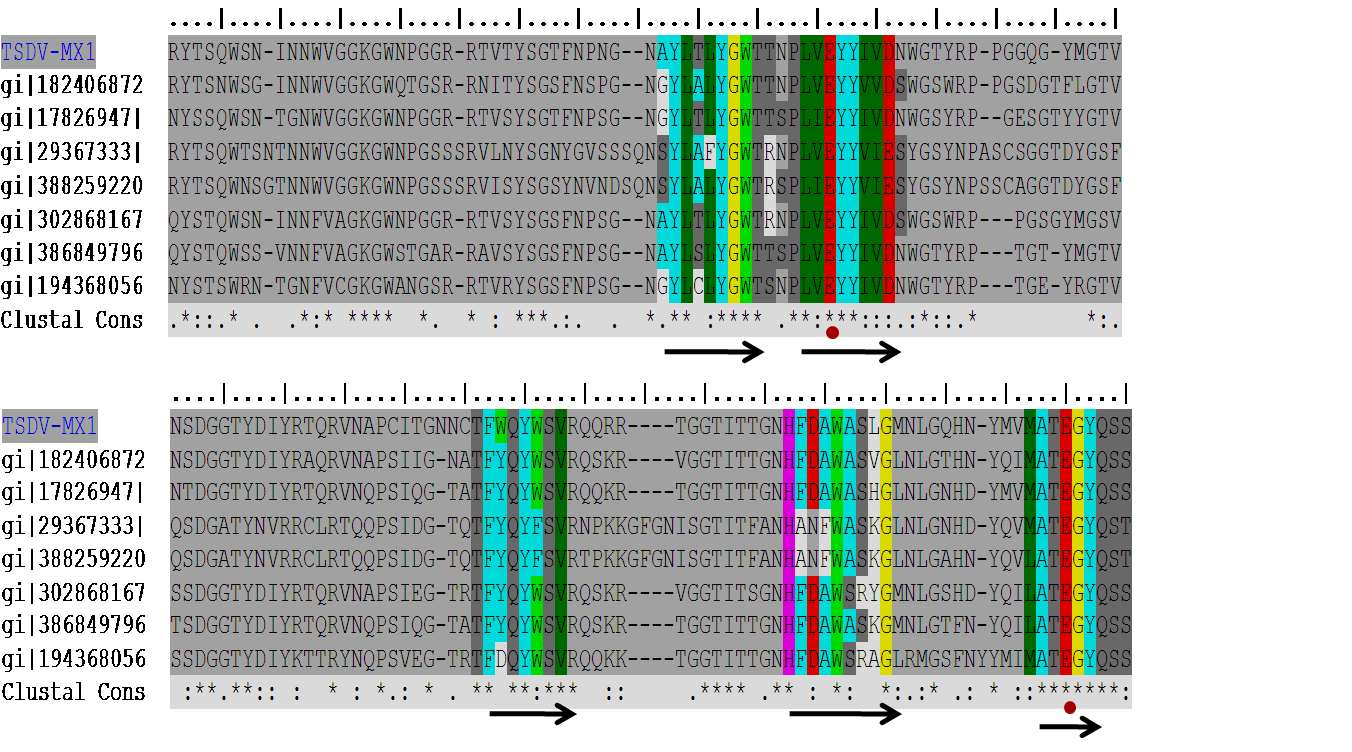


**Figure S2**

Supplement: Figure S2 — Multiple sequence alignment of xylanase with other xylanases available in database. GenBank accession number and source of microorganisms were given as follows: 182406872 (glycosyl hydrolase family 11 precursor [uncultured bacterium]),17826947 (Pseudomonas sp. ND137), 29367333 (uncultured Cellvibrio sp.), 388259220 (Cellvibrio sp. BR), 302868167 (Micromonospora aurantiaca ATCC 27029), 386849796 (Actinoplanes sp. SE50/110), 194368056 (Streptomyces sp. S27). Five signature sequences: I (AYLTLYGW) II (VEYYIVDN), III (FWQYWSV), IV (HFDAWASLG) and V(MATEGY) of GH11 family are coloured. The two catalytically important residues (Glu 117 and Glu 209) are marked with black circle. (DOC) [file pone.0052459.s002.doc]
